# Analysis of Fluid Balance as Predictor of Length of Assisted Mechanical Ventilation in Children Admitted to Pediatric Intensive Care Unit (PICU)

**DOI:** 10.1155/2022/2090323

**Published:** 2022-03-20

**Authors:** Praveen Unki, Sushma Save

**Affiliations:** ^1^Department of Pediatrics, Adichunchanagiri Institute of Medical Sciences, Mandya 571448, India; ^2^Department of Pediatrics, T.N.M.C and B.Y.L. Nair Charitable Hospital, Mumbai 400008, India

## Abstract

**Background:**

Ventilator-associated lung injury (VALI) is a devastating complication of assisted mechanical ventilation (AMV) and is one of the root causes of prolonged AMV. Many strategies were made to decrease the effect of the same. This study is conducted to determine the association of prolonged AMV with fluid balance and pediatric index of mortality 2 (PIM2) score.

**Methods:**

This prospective observational study was carried out in a PICU of a tertiary care centre over a period of 12 months. Patient's fluid balance was calculated by tabulating fluid input-output over initial 48 hours of AMV. The PIM2 score on admission was documented. The association between qualitative variables was assessed by a chi-square test. Comparison of quantitative data measured between cases with duration of AMV ≥ 7 days and <7 days was done using the Mann–Whitney *U* test. Correlation between quantitative data was done by using the Pearson product moment correlation.

**Results:**

Out of 40 patients, 27 patients who had ≥15% positive fluid balance required prolonged mechanical ventilation. Similarly, 27 patients with PIM2 score ≥ 5 required prolonged AMV. On applying the Pearson chi-square test, we found a significant association between positive fluid balance and prolonged mechanical ventilation (*P* value = 2.25 × 10^−7^ (<0.05)). Likewise, a statistically significant association was found between PIM2 score and prolonged ventilation (*P* value = 1.19 × 10^−5^ (<0.05)).

**Conclusion:**

There is a significant association of prolonged AMV with positive fluid balance (>15%) and PIM2 score (>5). By strict maintenance of fluid balance with appropriate intervention, the length of AMV and PICU stay can be decreased.

## 1. Introduction

Mechanical ventilation is a crucial component of intensive care unit (ICU) that helps in supporting and sustaining life of critically ill patients. Ventilator-associated lung injury (VALI) is an inevitable consequence of assisted mechanical ventilation (AMV) [1, 2], and to curtail the same in acute respiratory distress syndrome (ARDS), lung protective ventilation strategies have been developed, which has led to significant reduction in death rates [3–5]. Different strategies such as sedation and ventilator weaning protocols including spontaneous breathing trials and decreasing duration of AMV are under trial to decrease VALI [1, 6, 7]. Fluid balance is one of the cardinal outcome measures related to exposure time of AMV [8–10]. Defective oxygenation and extended AMV were seen in patients with cumulative balance higher than 15% of the body weight [11, 12]. Patients requiring AMV for more than 7 days were considered to have prolonged mechanical ventilation [1]. After gathering data corresponding to initial 48 hours of AMV, this study is trying to exhibit the association between fluid balance and prolonged mechanical ventilation if any and generalising the same to wider population range and, similarly, to study the association of prolonged mechanical ventilation with other variables such as pediatric index of mortality 2 (PIM2) score, hypoxic respiratory failure, cardiovascular failure, age and gender [13, 14].

## 2. Materials and Methods

This prospective observational study was conducted over a period of 12 months in the pediatric intensive care unit (PICU) of a tertiary care institution after obtaining approval from the institutional ethics committee. The study enrolled all patients admitted in the PICU of age 29 days to 12 years, who fulfill the inclusion criteria during the 12-month period from the initiation of the study excluding those children previously admitted in the PICU of any other hospital and referred from those hospitals. Study participants were enrolled for a period of 7 days. At the time of admission, the patients' clinical profile was recorded in a prefixed case record form consisting of age, sex, date of admission, provisional clinical diagnosis, PIM2 score, and organ system primarily involved. Information regarding results of blood investigations like arterial blood gas parameters (PaO2, base excess, FiO2) was collected from the hospital records. Recordings of total fluid given and total fluid output in the first 48 hours of mechanical ventilation were documented. Fluid balance was calculated by the difference between the total amount of fluid administered and the sum of all the losses experienced during the first 48 hours of AMV by using the formula (fluid input − fluid output)/weight × 100 [15, 16]. PIM2 score was calculated by tabulating the following information such as (1) whether the admission was emergency/elective (in case of surgeries), (2) any underlying premorbid conditions (cardiac arrest out of hospital, severe combined immune deficiency, leukaemia/lymphoma after the first induction, spontaneous cerebral haemorrhage from aneurysm or AV malformation, cardiomyopathy, myocarditis, hypoplastic left heart syndrome, HIV infection, and neurodegenerative disorder), (3) response of pupils to light, (4) mechanical ventilation at any time during the first hour in PICU, (5) systolic blood pressure, (6) base excess, and (7) FiO2/PaO2 ratio [17]. The associated factors for mortality were scrutinized with SPSS 17. PIM2 score was used as a tool to distinguish death and survival at a 99.8 cutoff with 95% confidence interval (CI).

PaO2/FiO2 ratio was measured, and if found <200 in the absence of cyanotic heart disease or left ventricular dysfunction, diagnosis of hypoxic respiratory failure was made [18]. Individual patients were evaluated for the presence of cardiovascular failure which is defined as arterial hypotension (<5th percentile for the age) or the need to use vasoactive drugs (dopamine, epinephrine, and norepinephrine at any dose) [19, 20]. Duration of mechanical ventilation, duration of PICU stay, and outcome were recorded subsequently and analysed with precision.

## 3. Statistical Analysis

Qualitative data such as nominal data included sex, provisional clinical diagnosis, organ system involved, cardiovascular failure, and hypoxic respiratory failure were represented in the form of frequency and percentage. The association between qualitative variables was assessed by the chi-square test, with continuity correction for all 2 × 2 tables and by Fisher's exact test for all 2 × 2 tables where the chi-square test was not valid due to small counts. In the presence of small counts in tables with more than two columns, adjacent column data was pooled and the chi-square test reapplied. Continuity correction was applied for all 2 × 2 tables after pooling of data. Fisher's exact test was applied for all 2 × 2 tables where *P* value of continuity correction was not valid due to small counts, in spite of pooling of data (e.g., association between duration of mechanical ventilation ≥ 7 days and organ system involved). Among qualitative data, ordinal data included PIM2 score and was represented using mean ± SD and median and IQR (interquartile range). Comparison of PIM2 measured between cases with duration of mechanical ventilation ≥ 7 days and <7 days was done using the Mann–Whitney test.

Quantitative data was represented using mean ± SD and median and IQR. Quantitative data included duration of mechanical ventilation, age, duration of PICU stay, and fluid balance. Comparison of quantitative data measured between cases with duration of mechanical ventilation ≥ 7 days and <7 days was done using the Mann–Whitney *U* test as all data failed the “normality” test. Correlation between quantitative data (duration of mechanical ventilation, age, duration of PICU stay, and fluid balance) was done by using the Pearson product moment correlation, as all the data passed the “Shapiro–Wilk normality test.” Binary logistic regression was used to assess predictors of duration of mechanical ventilation ≥ 7 days as dependent variables and fluid balance ≥ 15% and PIM2 score ≥ 5 as independent (predictor) variables. In view of the study being a prospective study, relative risk was calculated for various risk factors. Appropriate statistical software including but not restricted to MS Excel and PSPP version 1.0.1 was used for statistical analysis. An alpha value (*P* value) of ≤0.05 was used as the cutoff for statistical significance.

## 4. Results

This is a study of 40 participants of which infants (<1 year) were maximum (21, i.e., 52.50%). Males outnumbered females comprising 57.50% (23) of the study population. Maximum number of admissions (16) had central nervous system involvement followed by respiratory system involvement. Out of the 40 children admitted in PICU during the study period, 28 (70%) children had prolonged mechanical ventilation. 29 (72.5%) patients had positive fluid balance ≥ 15%, and 31 (77.5%) patients had PIM2 score ≥ 5. 27 patients who had ≥15% positive fluid balance required prolonged mechanical ventilation as shown in Table 1.

Similarly, 27 patients who had PIM2 score ≥ 5 required prolonged mechanical ventilation. The Pearson chi-square test was applied to test the significance of association between positive fluid balance and prolonged mechanical ventilation. *P* value was 2.25 × 10^−7^ which is <0.05 (Table 2) suggesting rejection of null hypothesis and statistically significant association between two variants. Similarly, on applying the Pearson chi-square test to find out the association between PIM2 score and prolonged ventilation, observed *P* value was 1.19 × 10^−5^ (<0.05) suggesting statistically significant association. However, association of other variables with prolonged mechanical ventilation was not statistically significant. The mean duration of PICU stay was prolonged in patients who required prolonged mechanical ventilation.

## 5. Discussion

Child admitted to PICU may require mechanical ventilation as a part of therapy for one or more reasons, for short or prolonged duration depending on indication for ventilation and multiple other factors which directly or indirectly contribute to the disease process. AMV is a frequently used life support system which, in spite of its benefit, might cause damage if appropriate principles are not followed. Multiple factors play important roles in determining duration of ventilation such as fluid balance, PELOD (pediatric logistic organ dysfunction) score, PIM2 score on admission, underlying comorbidities, and cardiovascular and respiratory failure on admission [1, 2, 21, 22].

In our study which was done in a tertiary care setup including 40 study participants, maximum admissions (77.5%) were <5 yrs, with infants constituting 52.5%. Majority of admissions were also <5 yrs (31.36%) in the study done by Vidal et al. [1]. Among the study participants, 57.5% were males and the rest were females. Major admissions were due to central nervous system involvement (40%), followed by respiratory system diseases (26.75%). The overall patients who had prolonged mechanical ventilation admitted to our PICU were 70.0% (28). Similar results were observed in the study done by Khwannimit and Koonrangsesomboon [11], which was 72.8% (763/1048), and the study was conducted in a tertiary referral university teaching hospital in southern Thailand. In the study conducted by Vidal et al. [1], in a retrospective cohort of patients in the PICU of Hospital Italiano de Buenos Aires, 50.3% patients (82/163) had prolonged mechanical ventilation.

29 patients (72.5%) in this study had positive fluid balance of ≥15% and 28 patients (70%) had prolonged mechanical ventilation. 27 patients who had ≥15% positive fluid balance required prolonged mechanical ventilation as shown in Table 1. The Pearson chi-square test was applied to test the significance of association between positive fluid balance and prolonged mechanical ventilation. *P* value was 2.25 × 10^−7^ (<0.05) suggesting rejection of null hypothesis and statistical significance (Table 2).

In a study by Vidal et al. [1] on fluid balance and length of mechanical ventilation in children admitted to a single PICU, during the study period, 1655 patients were admitted; 249 remained on AMV for over 48 hours and 163 were included in the study. The univariate analysis showed that the age younger than 4 years old (OR 3.21, 95% CI: 1.38-7.48), respiratory disease (OR 4.94, 95% CI: 1.51-16.10), septic shock (OR 4.66, 95% CI: 1.10-19.65), PELOD > 10 (OR 2.44, 95% CI: 1.23-4.85), and positive balance > 13% of the body weight (OR 4.02, 95% CI: 1.08-15.02) were associated with prolonged mechanical ventilation.

In a study from a South African PICU by Ketharanathan et al. [23], one hundred consecutive PICU admissions were included; before PICU admission, 59 children received a median 8.0 (4.0–15.0) ml/kg of fluid bolus. During PICU admission, 40 patients received a median fluid bolus amount of 10.0 (3.0–27.5) ml/kg. A total of three patients had a fluid overload (FO)% ≥ 10, even after correction for convalescent weight. The median minimum and maximum amount of maintenance fluid during PICU admission was 60 (46.0–80.0) ml/kg/day and 100.0 (60.0–120.0)ml/kg/day, respectively. The PICU mortality was 8%, and 28-day mortality was 10%. Most of the patients with positive fluid balance had prolonged mechanical ventilation. Two of the three patients died with FO ≥ 10%. The prevalence of FO in a South African PICU was low, but higher FO% based on admission weight was significantly associated with 28-day mortality. In a study by Wiedemann et al., in a comparison of two fluid management strategies in acute lung injury, the rate of death at 60 days was 25.5% in the conservative-strategy group and 28.4% in the liberal-strategy group (*P* = 0.30; 95% confidence interval for the difference, −2.6 to 8.4%) [9]. The mean cumulative fluid balance during the first seven days was –136 ± 491 ml in the conservative-strategy group and 6992 ± 502 ml in the liberal-strategy group (*P* < 0.001). As compared with the liberal strategy, the conservative strategy improved the oxygenation index and the lung injury score and increased the number of ventilator-free days (14.6 ± 0.5 vs. 12.1 ± 0.5, *P* < 0.001) and days not spent in the intensive care unit (13.4 ± 0.4 vs. 11.2 ± 0.4, *P* < 0.001) during the first 28 days. Although there was no significant difference in the primary outcome of 60-day mortality, the conservative strategy of fluid management improved lung function and shortened the duration of mechanical ventilation and intensive care. These results support the use of a conservative strategy of fluid management in patients with acute lung injury.

In a study by van Mourik et al. [24], 600 ARDS patients were studied to know the predictability of fluid balance with 28-day and 90-day mortality along with length of ICU stay. 156 patients (26%) died within 28 days. Patients with a higher cumulative fluid balance on day 7 had a longer length of ICU stay and fewer ventilator-free days on day 28. Similar results were found for 90-day mortality. A more positive fluid balance predicted mortality, and a negative fluid balance showed a trend towards survival. Confounding factors that alter fluid balance such as hemodialysis and diuretic use have been considered separately. However, in our study, none of the patient required hemodialysis or diuretics.

In our study, out of 40 patients, 31 (77.5%) patients had PIM2 score ≥ 5 and 28 (70%) patients had prolonged mechanical ventilation. 27 patients who had PIM2 score ≥ 5 required prolonged mechanical ventilation and the odds ratio was 2.89 (95% confidence interval, 1.296-6.46). On applying the Pearson chi-square test, *P* value of 1.19 × 10^−5^ (<0.05) suggested statistically significant association between two variants. On applying Fisher's exact test, *P* value of 5.15 × 10^−5^ (<0.05) also indicates statistical significance as shown in Table 3. A prospective observation study was conducted by Gandhi et al. [21], with PIM2 score as an outcome predictor at a tertiary care PICU in India. Consecutive 119 patients were enrolled in the study, and PIM2 scoring was done for all patients. A patient with the worst PIM2 score had prolonged duration of ventilation with high mortality rate. The presence of shock was independently associated with mortality, as evidenced by binary logistic regression. Hypoxic respiratory failure (*P* value = 0.677) and cardiovascular failure (*P* value = 0.05) did not show any statistically significant association with prolonged AMV. In a multivariate retrospective cohort study conducted by Vidal et al. [1], out of 163 patients with prolonged AMV, 103 (63%) had hypoxic respiratory failure and 63 (38%) and 104 (63.8%) had cardiovascular failure.

On multivariate analysis of the variables involved in the study, by applying the Mann–Whitney *U* rank sum test, strong association of prolonged mechanical ventilation (≥7 days) was found with fluid balance ≥ 15%, PIM2 score, and duration of PICU stay. Correlation was found between duration of mechanical ventilation ≥ 7 days and fluid balance, PIM2 score ≥ 5, and duration of PICU stay with Pearson's *r* value 0.297, 0.034, and 0.925, respectively, as shown in Table 4. No association was found with respect to age, sex, hypoxic respiratory failure, and cardiovascular failure as shown in Table 5.

Our study emphasises the significant association of prolonged mechanical ventilation (duration ≥ 7 days) with positive fluid balance ≥ 15% and PIM2 score ≥ 5 in children admitted to PICU. Strict fluid input and output monitoring and adjusting fluid intake accordingly in the first 48 hrs significantly affect duration of AMV and outcome. Appropriate fluid management plays a crucial role in PICU especially in ventilated patients by limiting the lung injury there by oxygenation and final outcome. PIM2 score assessment helps in predicting the duration of ventilation and final outcome. However, there are other scoring systems such as PELOD which more precisely indicates the duration of ventilation. We would like to emphasise on the strict fluid input and output charting on admission and for whole duration of PICU stay, monitoring vitals and maintenance of normal oxygenation, because early recognition of excessive positive fluid balance, hypoxic respiratory failure, and cardiovascular failure and treating accordingly may alter the duration of mechanical ventilation and outcome of patients.

## 6. Limitations of the Study

We had calculated fluid balance for the initial 48 hrs of AMV after admitting to PICU, and we had not included the fluid balance for the remaining duration of mechanical ventilation. We measured PIM2 score, cardiovascular failure, and hypoxic respiratory failure on admission, but these variables can change during the period of mechanical ventilation and PICU stay, which can alter the duration of mechanical ventilation and outcome. Thus, we might have missed subsequent measurement of fluid balance which could have had implications on duration of ventilation. We have not included the final outcome as death or discharge. We have conducted the study in a tertiary care centre that acts as a referral centre for many peripheral hospitals. Thus, our PICU bears the brunt of the critically ill children from all over the city. The sample size included in the study is less than the expected because of the decreased number of admissions during the study period in comparison with previous years. Thus, the results may not be applied to the general population or other peripheral centre hospitals.

## 7. Conclusions

The study has demonstrated significant associations of fluid balance ≥ 15% and PIM2 score ≥ 5 with prolonged duration of mechanical ventilation. Patients who had positive fluid balance ≥ 15% had more chances of prolonged mechanical ventilation in comparison to those who had PIM2 score ≥ 5, indicating more significant association of positive fluid balance with duration of mechanical ventilation. We found less significant association with hepatobiliary and renal involvement; however, because of small sample size it cannot be applied to the general population. To establish a clear-cut association with organ system involvement and to strengthen the significance of association, further prospective studies are required with a large sample size.

## Figures and Tables

**Table 1:** Association among the cases between duration of mechanical ventilation ≥ 7 days and fluid balance (≥15%).

Duration of mechanical ventilation ≥7 days	Fluid balance (≥15%)		Total
	Yes	No	
Yes	27	1	28 (70%)
No	2	10	12 (30%)
Total	29 (72.5%)	11 (27.5%)	40

**Table 2:** Significance of association between positive fluid balance and prolonged mechanical ventilation.

Chi-square test	Value	df	*P* value	Association
Pearson chi-square test	26.804	1	2.25 × 10^−7^	Significant
Continuity correction	22.953	1	1.66 × 10^−6^	Significant
Fisher's exact test			8.05 × 10^−7^	Significant

df: degrees of freedom.

**Table 3:** Significance of association between PIM2 score and prolonged ventilation.

Chi-square test	Value	df	*P* value	Association
Pearson chi-square	19.177	1	1.19 × 10^−5^	Significant
Continuity correction	15.730	1	7.31 × 10^−5^	Significant
Fisher's exact test			5.15 × 10^−5^	Significant

df: degrees of freedom.

**Table 4:** Correlation between duration of mechanical ventilation (days) and various other variables.

Variables	Pearson correlation	Duration of mechanical ventilation	Age (yrs.)	Duration of PICU stay (days)	Fluid balance (%)	PIM2 score
Duration of mechanical ventilation (days)	Pearson's *r*	1.000	-0.224	0.925^∗∗^	0.297	0.034
	*P* value		0.165	0.01	0.063	0.833
Age (yrs.)	Pearson's *r*	-0.224	1.000	-0.175	-0.302	-0.031
	*P* value	0.165		0.280	0.059	0.852
Duration of PICU stay (days)	Pearson's *r*	0.925^∗∗^	-0.175	1.000	0.168	0.027
	*P* value	0.01	0.280		0.299	0.869
Fluid balance (%)	Pearson's *r*	0.297	-0.302	0.168	1.000	0.176
	*P* value	0.063	0.059	0.299		0.278
PIM2 score	Pearson's *r*	0.034	-0.031	0.027	0.176	1.000
	*P* value	0.833	0.852	0.869	0.278	

^∗∗^Correlation is significant at the 0.01 level (2-tailed).

**Table 5:** Comparison of various variables between cases with and without mechanical ventilation duration ≥ 7 days.

Variables	Mechanical ventilation duration ≥7 days	Mean	SD	Median	IQR	*Z* value	*P* value
Duration of PICU stay (days)^^^	Yes	17.839	11.519	14.000	12.800	-3.618	0.0003
	No	8.050	4.300	7.350	6.400	Difference is significant	
Fluid balance (%)	Yes	0.192	0.067	0.179	0.041	-3.217	0.00129
	No	0.100	0.094	0.090	0.090	Difference is significant	
PIM2 score^#^	Yes	10.828	8.049	9.190	5.800	-2.981	0.00287
	No	5.353	6.127	2.665	5.597	Difference is significant	

^^^Data failed “normality test.” Hence, Mann–Whitney *U* rank sum test applied. ^#^Ordinal data. Hence, Mann–Whitney *U* rank sum test applied. SD: standard deviation; IQR: interquartile range.

## Data Availability

The data used to support the findings of this study are included within the supplementary information file.

## Additional Points

*Peer Review*. The study was externally peer-reviewed.

## Supplementary Materials

Supplementary Materials Appendix 4: master chart.
