# Supplementary material for: Analysis of Fluid Balance as Predictor of Length of Assisted Mechanical Ventilation in Children Admitted to Pediatric Intensive Care Unit (PICU)
Source: Int J Pediatr. 2022 Mar 20;2022:2090323. doi: 10.1155/2022/2090323 (PMC8958081; doi:10.1155/2022/2090323)
Supplement: Supplementary Materials — Appendix 4: master chart. [file 2090323.f1.docx]

**Appendix 4: Master Chart**

| Sl. No | Sex | Age (yrs) | PIM 2 Score | Organ System Involved | Hypoxic  Respiratory Failure | Fluid Balance | Mechanical  Ventilation (days) | Duration of  IPCU  stay(days) | Diagnosis | Cardiovascular Failure |
| --- | --- | --- | --- | --- | --- | --- | --- | --- | --- | --- |
| 1 | Male | 2.1 | 0.116 | Central  Nervous System | Yes | 2.36% | 2.2 | 7.5 | Tubercular Meningitis | No |
| 2 | Male | 0.2 | 13.14 | Gastrointestina l | Yes | 26.40% | 7.3 | 9.7 | Acute gastroenteritis | No |
| 3 | Male | 0.4 | 24.58 | Respiratory System | Yes | 16% | 12.1 | 27 | Pneumonia | Yes |
| 4 | Male | 1.5 | 4.69 | Respiratory System | No | 30.50% | 4.3 | 4.5 | Pneumonia | No |
| 5 | Female | 10 | 1.71 | Central  Nervous System | No | -3.20% | 2.3 | 12 | Tubercular Meningitis | No |
| 6 | Male | 0.2 | 8.02 | Gastrointestina l | Yes | 10.50% | 3 | 8 | Acute gastroenteritis | Yes |
| 7 | Male | 0.3 | 6.4 | Hepatobiliary | No | 18.27% | 9 | 9 | Neonatal Cholestasis | No |
| 8 | Female | 9 | 44.5 | Renal | Yes | 16.94% | 7.2 | 9 | Disease with  Hypertensive Emergency | No |
| 9 | Male | 10 | 6.607 | Central  Nervous System | No | 18.70% | 9 | 9 | SubAcute Sclerosing Panencephalitis | Yes |
| 10 | Male | 0.2 | 10.92 | Cardiovascular system | Yes | 17.14% | 23 | 23 | Disease with Lower  respiratory tract infection | Yes |

| Sl. No | Sex | Age (yrs) | PIM 2  Score | Organ System Involved | Hypoxic  Respiratory Failure | Fluid Balance | Mechanical  Ventilation(da ys) | Duration of  IPCU  stay(days) | Diagnosis | Cardiovascular Failure |
| --- | --- | --- | --- | --- | --- | --- | --- | --- | --- | --- |
| 11 | Male | 3.5 | 9.86 | Central Nervous System | No | 18.90% | 23 | 23 | Ataxia | No |
| 12 | Female | 0.7 | 5.84 | Gastrointestin  al | No | 15.30% | 18 | 18 | Acute gastroenteritis | Yes |
| 13 | Male | 0.1 | 2.27 | Central  Nervous System | No | 0.67% | 3.3 | 15 | Meningitis in status epilepticus | No |
| 14 | Male | 0.3 | 6.35 | Gastrointestin al | No | 19.90% | 58 | 58 | Acute gastroenteritis | Yes |
| 15 | Male | 0.6 | 11.75 | Cardiovascular system | Yes | 16.69% | 13 | 13 | Acyanotic Congenital Heart Disease | Yes |
| 16 | Female | 10 | 9.08 | Hepatobiliary | No | 15.50% | 12.6 | 12.6 | Acute liver Failure | No |
| 17 | Male | 0.1 | 0.97 | Central  Nervous System | No | 8.40% | 3.6 | 4.6 | Acute Bacterial Meningitis | No |
| 18 | Female | 0.2 | 9.3 | Cardiovascular system | Yes | 18.17% | 30 | 30 | Acyanotic congenital heart disease | Yes |
| 19 | Female | 0.3 | 10.8 | Gastrointestin al | Yes | 21.60% | 7.1 | 7.1 | Acute gastroenteritis | Yes |
| 20 | Female | 11 | 1.76 | Central  Nervous System | No | 17.34% | 9.1 | 11.5 | Left Hemiplegic Cerebral palsy with right sided weakness | No |

| Sl. No | Sex | Age (yrs) | PIM 2 Score | Organ System Involved | Hypoxic Respiratory Failure | Fluid Balance | Duration of Mechanical Ventilation(days) | Duration of IPCU stay(days) | Diagnosis | Cardiovascular Failure |
| --- | --- | --- | --- | --- | --- | --- | --- | --- | --- | --- |
| 21 | Male | 4 | 12.92 | Gastrointestinal | Yes | 15.00% | 8.6 | 8.6 | Septic Arthritis with intestinal obstruction | No |
| 22 | Male | 1.1 | 7.4 | Central Nervous System | No | 19.81% | 11.2 | 11.2 | Encephalitis with Femur fracture | No |
| 23 | Female | 2 | 6.6 | Respiratory System | Yes | 23.00% | 18 | 23 | Pneumonia | No |
| 24 | Male | 0.9 | 21.76 | Respiratory System | Yes | 11.93% | 4.7 | 4.8 | Pneumonia | No |
| 25 | Male | 0.2 | 19.29 | Respiratory System | Yes | 16.48% | 11.4 | 11.4 | Pneumonia | Yes |
| 26 | Male | 0.3 | 5.91 | Central Nervous System | No | 17.60% | 19.4 | 22.1 | Hypoxic ischaemic  encephalopathy with Epilepsy | No |
| 27 | Female | 0.1 | 14.5 | Respiratory System | Yes | 20.00% | 9.3 | 20.2 | pneumonia | No |
| 28 | Female | 0.4 | 10.08 | Central Nervous System | No | 20.89% | 8.8 | 10 | Intracranial Hemorrhage | No |
| 29 | Male | 0.1 | 11.79 | Infection | Yes | 12.00% | 2.1 | 3 | Sepsis | No |
| 30 | Male | 0.3 | 12.3 | Respiratory System | Yes | 22.90% | 36.2 | 46 | Pneumonia | No |

| Sl. No | Sex | Age (yrs) | PIM 2  Score | Organ System Involved | Hypoxic Respiratory Failure | Fluid Balance | Duration of Mechanical Ventilation (days) | Duration of IPCU stay(days) | Diagnosis | Cardiovascular Failure |
| --- | --- | --- | --- | --- | --- | --- | --- | --- | --- | --- |
| 31 | Male | 1.2 | 5.516 | central Nervous System | No | 24.00% | 2.6 | 7.2 | HIV Encephalopathy with Septic Shock | No |
| 32 | Female | 1 | 11.4 | Cardiovascular system | No | 16.82% | 8.1 | 10 | Pneumonia with Cyanotic Congenital heart disease | Yes |
| 33 | Female | 9 | 2.06 | Central Nervous System | No | 4.70% | 4 | 8 | Intracranial Hemorrhage with thrombocytopenia | No |
| 34 | Female | 0.1 | 7.25 | Gastrointestinal | No | 44.00% | 13 | 13 | Acute gastroenteritis | Yes |
| 35 | Female | 8 | 2.88 | Cardiovascular system | Yes | 9.30% | 4.2 | 16.5 | Denue shock syndrome | No |
| 36 | Male | 0.2 | 5.18 | Central Nervous System | No | 30.00% | 16.9 | 16.9 | Hydrocephalus with Raised Intracranial pressure | No |
| 37 | Female | 3 | 6.12 | Central Nervous System | No | 3.10% | 15.2 | 15.2 | Acute Necrotising Encephalopathy | No |
| 38 | Female | 6 | 7.59 | Central Nervous System | No | 15.00% | 8.2 | 17 | Meningoencephalitis | No |
| 39 | Male | 1.5 | 2.45 | Respiratory System | Yes | 8.60% | 2.9 | 5.5 | Pneumonia | No |
| 40 | Female | 7 | 5.77 | Central Nervous  System | No | 16.78% | 11.3 | 15 | Multiple intracranial Tuberculoma | No |
